# Maternal obesity in Africa: a systematic review and meta-analysis

**DOI:** 10.1093/pubmed/fdv138

**Published:** 2016-10-17

**Authors:** Ojochenemi J. Onubi, Debbi Marais, Lorna Aucott, Friday Okonofua, Amudha S. Poobalan

**Affiliations:** 1 Institute of Applied Health Sciences, University of Aberdeen, Foresterhill, Aberdeen AB25 2ZD, UK; 2 Department of Obstetrics and Gynaecology, College of Medical Sciences, University of Benin, Benin City, Edo State, Nigeria

**Keywords:** obesity, population-based and preventative services, pregnancy and childbirth disorders

## Abstract

**Background:**

Maternal obesity is emerging as a public health problem, recently highlighted together with maternal under-nutrition as a ‘double burden’, especially in African countries undergoing social and economic transition. This systematic review was conducted to investigate the current evidence on maternal obesity in Africa.

**Methods:**

MEDLINE, EMBASE, Scopus, CINAHL and PsycINFO were searched (up to August 2014) and identified 29 studies. Prevalence, associations with socio-demographic factors, labour, child and maternal consequences of maternal obesity were assessed. Pooled risk ratios comparing obese and non-obese groups were calculated.

**Results:**

Prevalence of maternal obesity across Africa ranged from 6.5 to 50.7%, with older and multiparous mothers more likely to be obese. Obese mothers had increased risks of adverse labour, child and maternal outcomes. However, non-obese mothers were more likely to have low-birthweight babies. The differences in measurement and timing of assessment of maternal obesity were found across studies. No studies were identified either on the knowledge or attitudes of pregnant women towards maternal obesity; or on interventions for obese pregnant women.

**Conclusions:**

These results show that Africa's levels of maternal obesity are already having significant adverse effects. Culturally adaptable/sensitive interventions should be developed while monitoring to avoid undesired side effects.

## Introduction

Obesity is a worldwide epidemic.^1^ Prevalence is higher in wealthy countries,^2,3^ but increasing in developing countries,^3,4^ with severe consequences.^5–8^

Pregnancy is a recognized obesity trigger.^9^ Maternal obesity incidence is increasing worldwide^10–17^ and associated with short- and long-term complications for mothers^14,18–22^ and children^23–26^ during pregnancy, delivery and post-delivery.

Many developing countries now experience a double burden of malnutrition^4,27–29^ with increased maternal overweight and obesity.^12,30^ In Africa, the obesity increase in women has been steeper than in Asia with more than 40% reproductive aged women being overweight or obese.^12^ In addition, sub-Saharan Africa has the highest global neonatal mortality rate^31^ but the slowest progress in reducing maternal mortality.^32^ Approximately, one in four maternal deaths results from pre-existing medical conditions including obesity and diabetes.^32,33^

Maternal obesity is assessed differently worldwide, but pre-pregnancy or first trimester body mass index (BMI) is widely recommended.^34–36^ Other measures include weight and mid-arm circumference.^37–39^ There is also a lack of consensus on recommendations for managing obese pregnant women but in any case guidelines that do exist may not be applicable to countries with inadequate healthcare services.^40,41^

While maternal under-nutrition effects are well known, there is a paucity of data on maternal obesity in African countries.^23,42,43^ A scoping exercise identified one study utilizing pooled data from demographic health surveys in sub-Saharan Africa focussing on maternal obesity effects on neonatal death.^44^ This indicates a need for a comprehensive literature review to assess the prevalence and burden of maternal obesity in Africa in order to develop policies and interventions to improve maternal health.^3,33^

The aim of this systematic review was to investigate maternal obesity in Africa assessing prevalence, socio-demographic associations, adverse pregnancy outcomes in mother and child, existing interventions along with knowledge and attitudes of pregnant women, and healthcare providers towards maternal obesity.

## Methodology

A comprehensive search of MEDLINE, EMBASE, Scopus, CINAHL and PsycINFO was conducted in August 2014 with no restriction on language or publication year (Supplementary data, Table S1). Google Scholar and references of relevant articles were also searched. Mesh terms and keywords for maternal obesity and geographical location were combined using Boolean operators. Studies, irrespective of design, conducted in Africa recording maternal obesity either by maternal BMI or by other weight measures at any time during pregnancy or immediately after delivery were included. All maternal and child outcomes with at least one obese group and a comparison group were assessed. Intervention studies to increase maternal weight, studies conducted in non-pregnant women and studies that targeted women with specific disease conditions such as HIV were excluded (Supplementary data, Table S2).

A data extraction form was developed and piloted, and quality assessment carried out using the Effective Public Health Practice Project quality assessment tool.^45^ Study quality was graded strong, moderate or weak based on selection bias, study design, confounders, data collection method and dropouts.^45^

All studies reporting maternal obesity prevalence in some way were included in prevalence comparisons using their criteria. Although BMI was preferred some studies reported more than one obesity measure. Maternal obesity was measured at different time points in different studies, while two studies adjusted BMI for gestational age. Where obesity was measured at different points in pregnancy in the same study, the earliest measurement was used. ‘Obese’ groups were mainly defined as BMI ≥30 kg/m^2^ with the comparator ‘non-obese’ group being a combination of overweight and normal weight participants. This was possible for all studies reporting BMI except one,^46^ where overweight and obese data were presented separately for prevalence but not for other outcomes, so this study was excluded from meta-analysis. One study^47^ measured obesity as BMI 27.6–41.8 kg/m^2^, another study^48^ as ≥28 kg/m^2^ and four other studies used weight ≥80 kg^49,50^ and ≥90 kg^51,52^ as obesity cut-off points. The results from these studies were included in meta-analysis for the respective outcome(s) measured.

### Pooling of evidence using meta-analyses

Raw data within obese and non-obese groups were extracted for each outcome. ‘Obese’ group sizes were sufficient making relative risks (RRs) appropriate for dichotomous outcomes and the mean difference (MD) for continuous measures with their 95% confidence interval. Pooled estimates for each outcome were calculated using meta-analysis where appropriate (two or more studies, similarly measured). Otherwise, results were described along with their individual estimated effects, subject to sufficient information and put in context.

A Health Technology Assessment report highlighted a lack of consensus on the important outcomes of maternal obesity,^14^ so this review assessed all outcomes reported within the included studies. Separate meta-analyses were considered for each using Review Manager Software (version 5.2) with heterogeneity assessed by the chi-squared and the *I*^2^ statistic. Fixed effect models were used unless otherwise indicated but for outcomes with moderate heterogeneity (*I*^2^ >50%, *P* ≥ 0.10), random effects models (REM) were used. In cases of substantial heterogeneity (*I*^2^ >75%), possible causes were explored and sensitivity analyses performed resulting in some studies being excluded from meta-analyses due to heterogeneity.^53^ Given the number of outcomes, only forest plots of outcomes meeting at least two of three criteria are presented in the online document (Supplementary data, Figs S1–9): those assessed by a large number of studies (≥5), with highly significant results and being a significant cause of maternal or child mortality in Africa.^31,33^

## Results of the literature search

**Fig. 1:**
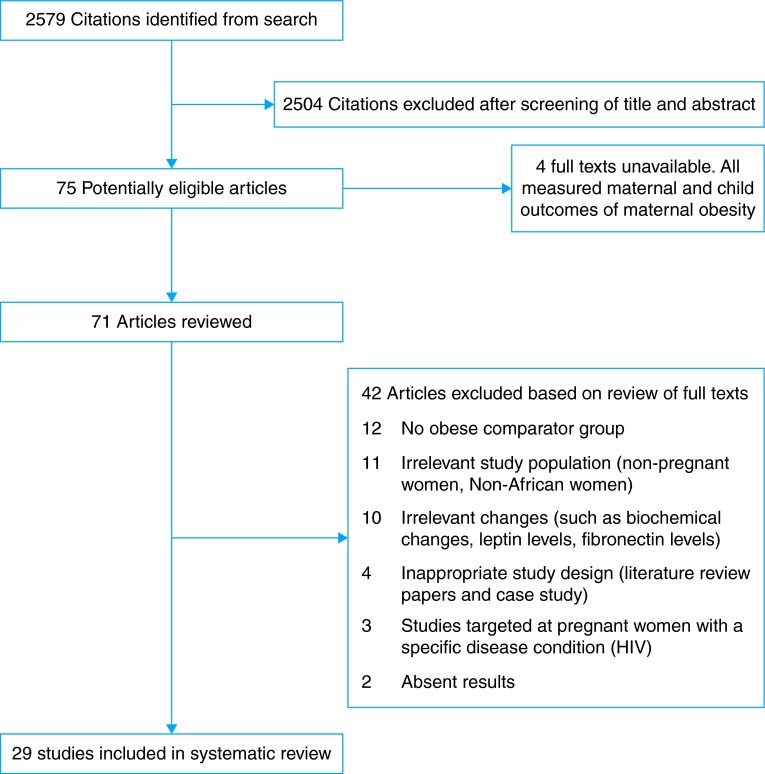
Flow diagram of the selection process for the review.

A total of 2579 titles and abstracts were identified. Initially, 300 were screened by two independent reviewers (O.J.O. and A.S.P.) refereed by a third reviewer (D.M.). Consistency established, the remainder were scanned by one reviewer (O.J.O.). Full texts for 75 potentially eligible papers were retrieved except for four studies (Fig. 1). After reading the full texts, 29 studies were included in this review, basic characteristics of which are presented in Table 1.

**Table 1:** Basic characteristics of included studies

*Author, year, country, study quality*	*Study type*	*Sample size*	*Outcomes*	*Enrolment dates*	*Obesity measure*	*Gestational age*
Efiong, 1975^49^ Nigeria Strong	Cohort	200	Labour outcomes: Prolonged labour, precipitate labour, caesarean section, cephalopelvic disproportion Child outcomes: Macrosomia (>4 kg), stillbirth, low birthweight, death Maternal outcomes: Pre-eclampsia, urinary tract infection, malpresentation, antepartum haemorrhage, postpartum haemorrhage, retained placenta, death	February 1972 to January 1975	Weight >80 kg	14th week of pregnancy
Lawoyin, 1993^54^ Nigeria Moderate	Cohort	492	Other outcomes: Pre-pregnancy weight	Not specified	BMI^a^	Delivery
Khan, 1996^55^ Egypt Moderate	Cohort	80	Prevalence Socio-demographic outcomes: Age, parity	1983–1985	BMI	Pre-pregnancy
Mahomed, 1998^47^ Zimbabwe Strong	Case control	338	Maternal outcome: Pre-eclampsia	June 1995 to April 1996	BMI	Immediately post-delivery
Olayemi, 2002^50^ Nigeria Moderate	Cross-sectional	3104	Prevalence Socio-demographic outcomes: Parity, marital status, ethnicity Labour outcomes: Cephalopelvic disproportion, prolonged labour, shoulder dystocia, retained placenta, genital laceration, caesarean section Child outcomes: Macrosomia (>4 kg), low birthweight, asphyxia (1 min) Maternal outcomes: Pre-eclampsia, gestational diabetes mellitus, urinary tract infection, anaemia, antepartum haemorrhage, malpresentation, thromboembolic disease, preterm gestation, post-term gestation, wound infection, postpartum haemorrhage, eclampsia, death	1 January 1995 to 31 December 1999	Weight ≥90 kg	Last antenatal visit before delivery
Adesina, 2003^51^ Nigeria Weak	Case control	190	Child outcome: Macrosomia (≥4 kg)	1 January 1998 to 31 December 2000	Weight >90 kg	Term
Anorlu, 2005^52^ Nigeria Moderate	Case control	368	Maternal outcome: Pre-eclampsia	February 2001 to August 2002	Weight ≥80 kg	Pre-pregnancy
Van Bogaert, 2005^56^ South Africa Weak	Cross-sectional	2042	Socio-demographic outcome: Parity	Not specified	Ponderal index	End pregnancy
Edomwonyi, 2006^57^ Nigeria Weak	Cross-sectional	300	Prevalence Socio-demographic outcome: Age Labour outcomes: Caesarean section, type of caesarean section, intra-operative complications	June 2004 to June 2005	BMI	Not stated
Villamor, 2006^58^ Tanzania Strong	Interrupted time series	73 689	Prevalence Socio-demographic outcomes: Parity, education, cohabitation, employment	1995–2004	BMI	≤14 weeks and <28 weeks
Adebami, 2007^59^ Nigeria Weak	Cross-sectional	473	Prevalence Child outcome: Foetal malnutrition	January to August 2001	BMI	Likely third trimester
Mamabolo, 2007^60^ South Africa Moderate	Cross-sectional	262	Prevalence Socio-demographic outcomes: Age, parity	May–August 1999 and February–April 2000	BMI	Third trimester (28–36 weeks)
Ward, 2007^61^ South Africa Moderate	Cohort	98	Socio-demographic outcomes: Age Maternal outcomes: Haemoglobin levels	Not specified	BMI	Pre-pregnancy
Abdul, 2009^62^ Nigeria Weak	Case control	425	Child outcomes: Macrosomia (≥4 kg)	January 2001 to December 2005	BMI	Booking
Kamanu, 2009^48^ Nigeria Weak	Cross-sectional	9040	Child outcomes: Macrosomia (>4.5 kg)	1 January 1999 to 31 December 2003	BMI Weight	Third trimester before delivery
Ngoga, 2009^63^ South Africa Weak	Case control	309	Socio-demographic outcomes: Parity Labour outcomes: Induction of labour, epidural during labour, instrumental delivery, episiotomy, perineal tear, caesarean section, wound sepsis Child outcomes: Macrosomia (>4.5 kg), asphyxia (<7 at 5 min), neonatal death Maternal outcomes: Urinary tract infections, anaemia	Not specified	BMI	Booking
Addo, 2010^46^ Ghana Weak	Retrospective cohort	1755	Prevalence Socio-demographic outcome: Age	1 January to 31 December 1992	BMI	First trimester
Basu, 2010^64^ South Africa Moderate	Cross-sectional	767	Prevalence Labour outcomes: Preterm labour, preterm rupture of membranes, induction of labour, caesarean section, failed induction of labour, longitudinal skin incision Maternal outcomes: Pregnancy-induced hypertension, gestational diabetes mellitus, urinary tract infection	February and September 2006	BMI	Booking (median 28 weeks)
Ugwuja, 2010^65^ Nigeria Weak	Cohort	349	Socio-economic outcomes: Age, parity, education, living accommodation Labour outcomes: Instrumental delivery, caesarean section, preterm delivery Child outcomes: Low birthweight, stillbirth Maternal outcomes: Anaemia, post-term delivery	Not specified	BMI	At recruitment (≤25 weeks)
Adesina, 2011^66^ Nigeria Moderate	Matched case control	236	Socio-demographic outcomes: Age, education, marital status, ethnicity, parity, social status Child outcomes: Low birthweight, macrosomia (≥4.2 kg), perinatal asphyxia (5 min APGAR <7), birth trauma, neonatal admission. Maternal outcomes: Hypertension in pregnancy, gestational diabetes, infection, pre-eclampsia, gestational age at delivery, assisted vaginal delivery, caesarean section, cephalopelvic disproportion, perineal laceration, postpartum haemorrhage, prolonged/obstructed labour	Not specified	BMI	≤32 weeks
Chigbu, 2011^67^ Nigeria Moderate	Cross-sectional	3167	Prevalence Socio-demographic outcomes: Age, parity, employment status, educational level, rural/urban residence Other outcomes: Pre-pregnancy weight	April 2009 to January 2010	BMI	First trimester (Mean 11.0 ±2.2 weeks)
Ezeanochie, 2011^68^ Nigeria Moderate	Cross-sectional Then Case control	2086 402-case control	Prevalence Socio-demographic outcomes: Age, parity, social class, marital status Maternal outcomes: Gestational diabetes, pregnancy-induced hypertension, antenatal admission, birth before 37 weeks, birth before 34 weeks Labour outcomes: Augmentation of labour, mode of delivery (spontaneous vaginal delivery, instrumental delivery, caesarean section), episiotomy, perineal tear, postpartum haemorrhage, maternal mortality. Child outcomes: sex, low birthweight, macrosomia (not specified), stillbirth, admission into neonatal special care unit, severe birth asphyxia (5 min APGAR <3)	January 2006 to December 2008	BMI	Booking
Jeremiah, 2011^69^ Nigeria Weak	Cohort	300	Prevalence Socio-demographic outcomes: Age, parity, educational status Maternal outcomes: antepartum haemorrhage, anaemia, malaria, urinary tract infection, sickle cell disease, malpresentation, twin gestation, preterm delivery, prolonged pregnancy, maternal death, gestational DM Labour outcomes: Caesarean section, genital lacerations or episiotomies, postpartum haemorrhage, wound infection Child outcomes: Macrosomia (≥4 kg), intrauterine foetal death, birth asphyxia (1 min APGAR), birth trauma, congenital abnormality, admission to special baby care unit, perinatal mortality	May 2006 and April 2007	BMI	Booking
Davies, 2012^70^ South Africa Strong	Data from previous cluster-RCT	1145	Prevalence Socio-demographic outcomes: Age, parity, education, marital status, employment, income, housing type	2009–2010	BMI	Adjusted BMI for gestational age.
El-Makhangy, 2012^71^ Egypt Moderate	Prospective cohort	250	Maternal outcome: Pre-eclampsia	Not specified	BMI	At 20 weeks and 28 weeks of gestation
Okafor, 2012^72^ Nigeria Weak	Cross-sectional	250	Prevalence	May 2008 to December 2010	BMI	Last weight before delivery
Koyanagi, 2013^73^ 7 countries Moderate	Cross-sectional	78 545	Prevalence Child outcome: Macrosomia (≥4 kg)	2004–2005	BMI	Booking
Iyoke, 2013^74^ Nigeria Strong	Retrospective cohort	1806 648 (cohort)	Prevalence Socio-demographic outcomes: Occupation, educational status, marital status, residence Maternal outcomes: Premature rupture of membranes, pre-eclampsia/eclampsia, antepartum haemorrhage, gestational diabetes, caesarean section, postpartum haemorrhage Child outcomes: Macrosomia (not specified), severe birth asphyxia, newborn intensive care admission	1 January 2010 to 31 December 2011	BMI	First trimester
Davies, 2013^75^ South Africa Strong	Data from previous cluster-RCT	1058	Prevalence Maternal outcomes: Maternal death, caesarean section, maternal hospital stay, preterm labour, post-term labour, gestational diabetes, pregnancy-induced hypertension Child outcomes: Stillbirth, neonatal death, low birthweight, macrosomia (≥4.5 kg)	2009–2010	BMI	Adjusted BMI for gestational age

BMI, body mass index; RCT, randomized controlled trial.

^a^Calculated as weight in kilograms divided by height in metre squared.

Included studies were from Nigeria (*n* = 16), South Africa (*n* = 7), Egypt (*n* = 2), Ghana (*n* = 1), Tanzania (*n* = 1), Zimbabwe (*n* = 1) and a final study conducted in seven African countries. With respect to quality, 11 studies were weak,^46,48,51,56,57,59,62,63,65,69,72^ 12 moderate^50,52,54,55,60,61,64,66,67,68,71,73^ and 6 studies were classified as strong.^47,49,58,70,74,75^ Most studies gave weak descriptions about controlling for confounders and/or about data collection methods. All outcomes were extracted and categorized into four major groups—prevalence, socio-demographic characteristics, labour, and child and maternal outcomes (Supplementary data, Table S3).

## Results of the review

### Prevalence of maternal obesity

Prevalence of maternal obesity was assessed by 16 studies, ranging between 6.5 and 50.7% (Table 2). Fifteen studies used BMI, while one study used a weight cut-off point at ≥90 kg.^50^

**Table 2:** Prevalence of maternal obesity

*Study, year*	*Sample size*	*Country*	*Timing of measurement*	*Measure*	*Prevalence of Obesity*
Khan, 1996^55^	80	Egypt	Pre-pregnancy	BMI^a^ percentiles ≥95th percentile	10% (8/80)
Villamor, 2006^58^	4068	Tanzania (*2004 data chosen*)	First trimester (gestational week ≤14 weeks)	BMI ≥30	7.3% (298/4068)
Addo, 2010^46^	1755	Ghana	First trimester	BMI ≥30.1	17.9% (314/1755)
Chigbu, 2011^67^	3167	Nigeria	First trimester	BMI ≥30	10.7% (339/3167)
Iyoke, 2013^74^	1806	Nigeria	First trimester	BMI ≥30	17.9% (340/1806)
Basu, 2010^64^	767	South Africa	Antenatal booking (median 28 weeks)	BMI ≥30	44% (337/767)
Ezeanochei, 2011^68^	2086	Nigeria	Antenatal booking	BMI >30	9.6% (201/2086)
Jeremiah, 2011^69^	4832	Nigeria	Antenatal booking	BMI >30	7.4% (357/4832)
Koyanagi, 2013^73^	78 545	7 African countries	Antenatal booking	BMI ≥30	Algeria: 24.9% (3672/14 761) Angola: 9.6% (328/3424) DRC: 6.5% (544/8375) Kenya 20.3% (544/2680) Niger: 11.1% (888/8018) Nigeria: 31.7% (2415/7621) Uganda 13.0% (1367/10558)
Olayemi, 2002^50^	3104	Nigeria	Third trimester	Weight ≥90 kg	7.4% (230/3104)
Edomwonyi, 2006^57^	300	Nigeria	Third trimester	BMI >30	50.7% (152/300)
Mamabolo, 2007^60^	262	South Africa	Third trimester	BMI ≥30	24.05% (63/262)
Okafor, 2012^72^	250	Nigeria	Third trimester	BMI ≥35	14.8% (37/250)
Davies, 2012^70^	1145	South Africa	Adjusted BMI for gestational age (GBMI)	BMI >29 to <50	33.53% (384/1145)
Davies, 2013^75^	1058	South Africa	Adjusted BMI for gestational age (GBMI)	BMI ≥29	33.1% (350/1058)
Adebami, 2007^59^	465	Nigeria	Not stated	BMI >30	17.8% (83/465)

BMI, body mass index; DRC, Democratic Republic of Congo.

^a^Calculated as weight in kilograms divided by height in metre squared.

Studies using pre-pregnancy or first trimester measurements suggest obesity prevalence between 9.0 and 17.9%.^46,55,58,67,74^ Those using ‘booking’ dates (antenatal registration) imply 6.5–44.0%,^64,68,69,73^ while third trimester reports indicate prevalence between 14.0 and 50.7%.^50,57,60,72^ The study by Adebami *et al*.^59^ while not specifying gestational age, reported a prevalence of 17.8%. Two other studies using ‘BMI adjusted for gestational age’ reported obesity prevalence at 33.1 and 33.5%, respectively.^70,75^ The study with the highest prevalence was measured among pregnant women scheduled for caesarean section.^57^

Worth highlighting is the study^73^ assessing maternal obesity at booking in seven countries: obesity prevalence was estimated as 6.5% in the Democratic Republic of Congo up to 31.7% in Nigeria. Also, of note, maternal obesity (using BMI measured in first trimester) increased from 2.4 to 7.3% over a 9-year period in Tanzania.^58^

### Socio-demographic associations of maternal obesity

Eight socio-demographic outcomes were considered for separate meta-analyses. Obese mothers were significantly older than non-obese mothers (number of studies *n* = 5^55,57,60,65,68^; MD 2.67 years, 2.12–3.22). Five studies were excluded from meta-analysis on age due to varied data types,^58,63^ unavailable data^64,67^ and heterogeneity.^71^ Maternal obesity was significantly associated with increasing age in three of these studies^58,63,64^ but not in two.^67,71^ One study excluded for heterogeneity^71^ may have had different results, because the women were younger (20–30 years) than the other studies.

The RR of obesity was increased in multiparous women (*n* = 4,^50,65,68,69^ REM, RR 1.49, 1.19–1.87) but not significantly in mothers older than 35 years (*n* = 2,^68,69^ REM, RR 1.32, 0.96–1.82), married mothers (*n* = 4,^50,66,68,74^ RR 1.17, 1.00–1.38), HIV-infected mothers (*n* = 2,^69,70^ RR 0.93, 0.77–1.13), mothers with tertiary education (*n* = 4,^65,66,69,70^ REM, RR 1.21, 0.92–1.58) or employed mothers (*n* = 3,^65,70,74^ RR 0.95, 0.84–1.08).

Meta-analysis was not performed on seven studies investigating parity due to varied data types,^55,60^ different study group characteristics,^56,58^ unavailable data^64,67^ and heterogeneity.^70^ In these, obesity was positively associated with parity in three studies,^55,58,60^ but had no association in the other four.^56,64,67,70^ For one study excluded from meta-analysis on marital status due to different study group characteristics,^58^ obesity was not associated with marital status. Three studies excluded from the tertiary education meta-analysis because of different study group characteristics,^58^ unavailable data^67^ and heterogeneity^74^ showed one positive relationship between maternal education and obesity^58^ another negative relationship,^74^ while the third study showed no relationship between maternal obesity and education.^67^ Out of two studies not included in the unemployment meta-analysis due to different study group characteristics^58^ and unavailable data,^67^ one found that employed women were more likely to be obese,^58^ while the other reported no relationship between maternal obesity and employment.^67^

Although two studies^67,74^ investigated obesity and urban dwelling, one did not provide data.^67^ However, both reported that urban mothers^67,74^ were more likely to be obese than rural women. Similarly, although varied data types prevented meta-analysis for social class one study reported mothers in higher social classes were more likely to be obese^66^ and another showed no significant relationship.^68^ Although meta-analysis was also not conducted for ethnicity,^50,66^ because of multiple ethnic groups, individually neither showed significance between ethnicity and obesity.

Nine outcomes were measured by single studies. These indicated no significant association between maternal obesity and wealth,^70^ type of living accommodation,^65^ smoking (RR 0.81, 0.42–1.59),^70^ possession of identity document (RR 1.02, 0.98–1.06),^70^ booked antenatal clinic (RR 1.00, 0.93–1.07),^70^ income (RR 0.90, 0.78–1.03),^70^ formal housing (RR 0.90, 0.74–1.08),^70^ electricity (RR 1.00, 0.95–1.04)^70^ or positive tuberculosis infection (RR 1.83, 0.26–12.96).^70^

### Effects of maternal obesity on labour outcomes

Meta-analysis was conducted on six labour outcomes (Supplementary data, Table S4). Obese mothers had increased risks of caesarean section (*n* = 8,^49,50,65,66,68,69,74,75^ RR 1.87, 1.64–2.12) (Supplementary data, Fig. S1) and instrumental delivery (*n* = 8,^49,50,63–66,68,69^ REM, RR 2.72, 1.29–5.72) (Supplementary data, Fig. S2). Two studies were excluded from the caesarean section meta-analysis due to heterogeneity.^63,64^ In one, there was no relationship between obesity and caesarean section,^64^ while in the other, morbidly obese mothers (BMI ≥40 kg/m^2^) had significantly increased caesarean section rates compared with non-obese mothers (20–25 kg/m^2^).^63^ In the only study^57^ categorizing caesarean section into elective and emergency, obese mothers had marginally increased risks of elective caesarean section (RR 1.22, 1.01–1.47) and reduced risks of emergency caesarean section (RR 0.74, 0.56–0.99).

Overall, there was no significant relationship between maternal obesity and induction of labour, prolonged labour, episiotomy/perineal tear or cephalopelvic disproportion. However, one study (excluded from meta-analysis based on heterogeneity) reported significantly increased risks of induction of labour and perineal tear among morbidly obese mothers compared with non-obese mothers.^63^

Ten other labour outcomes reported in single studies indicate obese mothers more likely to have intra-operative complications (RR 2.92, 1.77–4.82)^57^ and less likely to have general anaesthesia (RR 0.32, 0.21–0.51) compared with non-obese mothers.^57^ Morbidly obese mothers were more likely to require epidural pain relief (RR 60.30, 3.63–1000.71).^63^ There was no significant relationship between maternal obesity and any indication for caesarean section,^57^ longitudinal skin incision (RR 1.32, 0.83–2.11),^64^ failed induction of labour (RR 2.25, 0.44–11.63),^64^ shoulder dystocia (RR 9.04, 0.49–166.91),^50^ precipitate labour (RR 0.50, 0.09–2.67),^49^ difficult laparotomy^64^ or difficult delivery of neonate during caesarean section.^64^

### Effects of maternal obesity on child outcomes

Several studies with sufficient homogeneity reported on seven child outcomes (Supplementary data, Table S5). Obese mothers had increased risks of macrosomia (*n* = 9,^49,51,62,63,68,69,73–75^ REM, RR 1.83, 1.51–2.21) (Supplementary data, Fig. S3) admission of neonate into special care baby or intensive care unit (*n* = 4,^66,68,69,74^ RR 1.56, 1.19–2.06), and reduced risks of low-birthweight babies (*n* = 6,^49,50,65,66,68,75^ RR 0.73, 0.53–0.99) (Supplementary data, Fig. S4) compared with non-obese mothers. Three studies excluded from the macrosomia meta-analysis based on heterogeneity, also showed positive relationships between maternal obesity and macrosomia.^48,50,66^ There was no significant association between maternal obesity and stillbirth, perinatal mortality, birth asphyxia using 5 and 1 min APGAR scores (Supplementary data, Fig. S5) or birth injuries.

Eight other child outcomes were assessed by single studies. In these maternal obesity was not associated with foetal malnutrition using the Clinical Assessment of Foetal Nutritional Status score (RR 0.88, 0.44–1.69)^59^ or congenital abnormality (RR 5.00, 0.24–103.28).^69^ However, obese mothers had increased risks of ‘baby stay for over 24 h in the hospital’ (RR 1.63, 1.25–2.13),^75^ increased number of days stayed in the hospital,^75^ and higher birthweight *z*-scores, birth length *z*-scores and head circumference *z*-scores.^75^ The risk of infant death (RR 0.28, 0.08–0.93)^75^ was significantly reduced among obese mothers.

### Effects of maternal obesity on maternal outcomes

Meta-analyses were possible on 17 maternal outcomes (Supplementary data, Table S6). Compared with normal weight women, obese mothers were found to have increased risks of wound infection (*n* = 3,^50,63,69^ RR 3.21, 1.28–8.06), gestational diabetes mellitus (*n* = 6,^64,66,68,69,74,75^ RR 2.42, 1.47–3.98) (Supplementary data, Fig. S6), pregnancy-induced hypertension (*n* = 3,^64,68,75^ REM, RR 1.59, 1.02–2.50), pre-eclampsia (*n* = 9,^47,49,50,52,63,66,69,71,74^ REM, RR 2.19, 1.58–3.03) (Supplementary data, Fig. S7), antepartum haemorrhage (*n* = 4,^49,50,69,74^ RR 3.67, 1.77–7.62) (Supplementary data, Fig. S8), postpartum haemorrhage (*n* = 6,^49,50,66,68,69,74^ RR 1.86, 1.18–2.92) (Supplementary data, Fig. S9), maternal hospital admission (*n* = 3,^63,68,75^ RR 1.38, 1.21–1.57), urinary tract infection (*n* = 5,^50,63,64,66,69^ RR 1.74, 1.05–2.88), postdate pregnancy (*n* = 6,^50,64–66,69,75^ RR 1.22, 1.01–1.47), malpresentation (*n* = 3,^49,50,69^ RR 3.01, 1.43–6.32), preterm rupture of membranes (*n* = 2,^64,74^ RR 2.88, 1.78–4.67) and pre-existing diabetes mellitus (*n* = 3,^50,63,65^ RR 2.98, 1.21–7.34). The risks of maternal anaemia were lower among obese mothers, although not significantly (*n* = 3,^50,65,69^ RR 0.90, 0.75–1.07). There were no significant associations between maternal obesity and maternal mortality, preterm labour, retained placenta and chronic or essential hypertension. Two studies excluded from meta-analyses based on heterogeneity reported significantly increased risks of pregnancy-induced hypertension in obese^66^ and morbidly obese mothers,^63^ and significantly lower risks of maternal anaemia among morbidly obese mothers^63^ compared with non-obese mothers.

Eleven maternal outcomes assessed by single studies indicate that obese mothers had significantly higher haemoglobin levels (MD 0.95 g/dl, 0.01–1.89),^61^ and morbidly obese mothers had longer gestation duration (MD 1.20 weeks, 0.76–1.64)^63^ than non-obese mothers. However, there was no association between maternal obesity and sickle cell anaemia (RR 0.33, 0.01–8.12),^69^ thromboembolic disease (RR 3.01, 0.12–73.57),^50^ eclampsia (RR 3.01, 0.12–73.57),^50^ glycosuria (RR 6.00, 0.74–48.94),^49^ twin gestation (RR 0.67, 0.11–3.93),^69^ hyperemesis gravidarum (RR 3.00, 0.12–72.77),^49^ malaria (RR 0.67, 0.11–3.93),^69^ miscarriages (RR 0.87, 0.38–2.00)^75^ or termination of a pregnancy (RR 1.23, 0.21–7.35).^75^

With respect to weight gain during pregnancy, one study showed that obese mothers were more likely to gain less weight than non-obese mothers (RR 0.60, 0.44–0.83).^49^ In another study,^74^ obese mothers had lower risks of gaining either excessive (RR 0.43, 0.30–0.60) or inadequate weight (RR 0.11, 0.06–0.20) based on the Institute of Medicine standards, compared with non-obese mothers. In yet another study, it was reported that weight gain in pregnancy was not significantly different between obese, overweight and normal weight women.^55^

## Discussion

### Main finding of this study

This review confirms maternal obesity as an emerging major public health issue in Africa, which is increasing in many African countries but varies between countries. When measured in the third trimester, obesity levels are high (up to 50.7%); however, even when measured in the first trimester, levels in Africa are comparable (up to 17.9%) with some developed countries, where approximately one in five pregnant women are obese.^76^

Obesity was measured at different time points by individual studies, using different measures and cut-offs points. Two studies used BMI adjusted for gestational age. From this review and other African studies,^43,77^ pregnant women generally register late for antenatal care, usually in the second trimester. In addition, only urban women with tertiary education knew their pre-pregnancy weights.^54,67^ Therefore, it may not ever be feasible to use pre-pregnancy or first trimester BMI,^14^ for diagnosing maternal obesity in these settings. Antenatal booking measurements were used by some studies, but due to varying times of booking across studies, these measurements may be heterogeneous. Several studies have investigated adjusting BMI for gestational age,^75,78^ BMI centile charts^79^ and weight gain charts,^43^ for assessing maternal obesity. It is also worth mentioning that apart from BMI, other anthropometric measurements such as maternal weight and mid-upper arm circumference are associated with adverse pregnancy outcomes.^38,43^ This warrants further exploration in order to reach a consensus on appropriate standardized measures for maternal obesity. Ideally, women should be encouraged to register early for antenatal care.

This review and others have identified several detrimental effects of obesity on both mother and child.^19,20,80,81^ The outcomes of maternal obesity could be viewed differently in terms of importance,^14^ both from a cultural and health system point of view. For example, many African women are averse to caesarean section^82^ and have poor access to safe caesarean section.^83^ Some adverse outcomes identified in this study such as haemorrhage and pre-eclampsia are important causes of maternal mortality in sub-Saharan Africa^84^ and hence might be even more critical in an African setting.

Among the socio-demographic associations, maternal obesity was found to be higher in older, multiparous women and among urban settlements in Africa but not associated with wealth. Obesity was previously seen as a disease of the affluent in developing settings, but recent evidence shows increasing obesity among both poor and rich.^85^

While addressing the issue of obesity in pregnant women, clinicians and researchers still need to be mindful of maternal under-nutrition. Preterm labour, low birthweight and anaemia were more common in non-obese pregnant women, although only low birthweight was significant in this review. Other studies have shown that preterm labour and anaemia are associated with underweight in pregnancy,^86,87^ while others^88^ found that maternal obesity was not independently associated with increased preterm deliveries. While high gestational weight gain might be helpful in preventing low birthweight, this must be balanced against other maternal and child health risks.^41^

Despite the broad and robust search strategy, this review did not identify any studies in Africa investigating knowledge and attitudes of healthcare professionals relating to maternal obesity let alone for mothers. It is crucial that health professionals and mothers are educated on issues of maternal obesity, as inadequate knowledge and poor attitudes of either group can create barriers to effective obesity interventions.^89–91^

### What is already known on this topic

Several observational studies have been conducted in Africa assessing the prevalence and/or associations between maternal obesity and various health outcomes.

### What this study adds

To our knowledge, this is the first comprehensive systematic review on maternal obesity in Africa. It highlights the high prevalence, problems with measurement and adverse effects of obesity for pregnant women across Africa. It also highlights the urgent need for exploratory study(s) with both mothers and healthcare professionals to assess their knowledge and perceptions towards maternal obesity. This will help to develop measurement tools, and tailor interventions incorporating all stakeholders' views for management of maternal obesity in African countries.

### Limitations of this study

For the various meta-analyses, the crude study estimates could have introduced bias due to confounding factor effects in the different studies. Owing to different maternal obesity definitions, there is potential for misclassification of obesity with possible overestimation or underestimation of effect sizes. Both pre-pregnancy obesity and excessive gestational weight gain are independently associated with poor pregnancy outcomes.^13,20,25,92^ This review did not differentiate between pre-pregnancy obesity and excessive gestational weight; therefore, the results should be interpreted in context. Additionally, these studies represent only a few African countries and may not generalize all African women. Finally, four full text articles not retrieved should none-the-less be considered while interpreting results. All four studies (Supplementary data, Table S2) found that maternal obesity in Africa was associated with adverse labour, child or maternal outcomes.

## Conclusion

Maternal obesity in Africa is of significant prevalence, with important adverse effects. Culturally adaptable/sensitive interventions should be developed while monitoring to avoid undesired side effects such as low birthweight.

## Supplementary data

Supplementary data are available at *PUBMED* online.

## Funding

This work was supported by the Commonwealth Scholarship Commission in the United Kingdom.

## Supplementary Material

Supplementary Data
