# Supplementary material for: Maternal obesity in Africa: a systematic review and meta-analysis
Source: J Public Health (Oxf). 2016 Oct 17;38(3):e218–31. doi: 10.1093/pubmed/fdv138 (PMC5072166; doi:10.1093/pubmed/fdv138)
Supplement: Supplementary Data [file supp_38_3_e218__index.html]

Maternal obesity in Africa: a systematic review and meta-analysis — Maternal obesity in Africa: a systematic review and meta-analysis — Supplementary Data 

# Maternal obesity in Africa: a systematic review and meta-analysis

## Supplementary Data

Supplementary Data

- Supplementary Data - Doc file
