# Supplementary material for: Maternal obesity in Africa: a systematic review and meta-analysis
Source: J Public Health (Oxf). 2016 Oct 17;38(3):e218–31. doi: 10.1093/pubmed/fdv138 (PMC5072166; doi:10.1093/pubmed/fdv138)

**Title: Maternal Obesity in Africa – A Systematic Review**

Authors: Ojochenemi J. Onubi, Debbi Marais, Lorna Aucott, Friday Okonofua, Amudha S. Poobalan

eTable 1: Search Strategy

eTable 2: Excluded and missing studies

eTable 3: Extracted outcomes from all included studies

eTable 4: Effects of maternal obesity on labour outcomes

eTable 5: Effects of maternal obesity on child outcomes

eTable 6: Effects of maternal obesity on maternal outcomes

eFigure 1: Caesarean section forest plot for obese and non-obese pregnant women

eFigure 2: Instrumental delivery forest plot for obese and non-obese pregnant women

eFigure 3: Macrosomia forest plot for obese and non-obese pregnant women

eFigure 4: Low birth weight forest plot for obese and non-obese pregnant women

eFigure 5: Birth Asphyxia forest plot for obese and non-obese pregnant women

eFigure 6: Gestational Diabetes forest plot for obese and non-obese pregnant women

eFigure 7: Pre-eclampsia forest plot for obese and non-obese pregnant women

eFigure 8: Antepartum Haemorrhage forest plot for obese and non-obese pregnant women

eFigure 9: Postpartum Haemorrhage forest plot for obese and non-obese pregnant women

**eTable 1**: Search Strategy

| **Number** | **Searches** |
| --- | --- |
| 1 | Maternal obesity/ |
| 2 | Obese parturients.mp. |
| 3 | (maternal adj5 (obesity or adiposity or weight gain or overweight)).mp. |
| 4 | (pregnan$ adj5 (obesity or adiposity or weight gain or overweight)).mp. |
| 5 | or/1-4 |
| 6 | "Africa south of the Sahara"/ or South Africa/ or Africa/ or North Africa/ or Central Africa/ |
| 7 | 5 and 6 |

**eTable 2: Table of Excluded studies and Missing studies**

| **Excluded Studies** | |
| --- | --- |
|  | Ahmadu BU, Gofama MM, Ashir GM, Ayub-Eniola AA, Abdulrahman M, Ambe JP. The effect of maternal pregnancy body mass index as a measure of pregnancy weight gain on neonatal birth weight in Maiduguri metropolitan council of Borno state, Nigeria. Greener Journal of medical Sciences 2012;2(6):168-172 |
|  | Adesina AF, Peterside O, Anochie I, Akani NA. Weight status of adolescents in secondary schools in port Harcourt using Body Mass Index (BMI). Italian Journal of Pediatrics. 2012;38(1). |
|  | Akpan JO. A comparison of maternal and cord blood glucose levels in diabetic and non-diabetic Nigerians in relation to birth weight and maternal body mass index. Acta Diabetol Lat 1989; 26(2):95-102. |
|  | Bamgbade OA, Khalaf WM, Ajai O, Sharma R, Chidambaram V, Madhavan G. Obstetric anaesthesia outcome in obese and non-obese parturients undergoing caesarean delivery: an observational study. International journal of obstetric anesthesia 2009; 18(3): 221-225. |
|  | Banda Y, Chapman V, Goldenberg RL, Chi BH, Vermund SH, Stringer JSA. Influence of body mass index on pregnancy outcomes among HIV-infected and HIV-uninfected Zambian women. Tropical Medicine and International Health 2007; 12(7):856-861. |
|  | Changamire FT, Mwiru RS, Peterson KE, et al. Effect of multivitamin supplements on weight gain during pregnancy among HIV-negative women in Tanzania. Maternal and Child Nutrition 2012: 1-14. |
|  | Colatrella A, Braucci S, Festa C, et al. Hypertensive disorders in normal/over-weight and obese type 2 diabetic pregnant women. Experimental and clinical endocrinology & diabetes: official journal, German Society of Endocrinology [and] German Diabetes Association 2009; 117(8): 373-377. |
|  | Cresswell JA, Campbell OM, De Silva MJ, Filippi V. Effect of maternal obesity on neonatal death in sub-Saharan Africa: multivariable analysis of 27 national datasets. Lancet 2012; 380(9850):1325-1330. |
|  | Ebirim NL, Lagiri B, Buowari YO. Case Report: Spinal Anaesthesia for Emergency Caesarean Section in a Morbid Obese Woman with Severe Preeclampsia. Case Reports in Anesthesiology 2012; 2012 (Aritcle ID 586235). |
|  | Ekaidem IE, Bolarin DM, Udoh AE, Etuk SJ, Udiong EJ. Plasma Fibronectin Concentration in obese/overweight pregnant women: a possible risk factor for preeclampsia. Indian Journal of Clinical Biochemistry 2011; 26(2):187-192. |
|  | El Hiday MM, Zumrawi FY. The effect of a nutrition education programme on pregnant women attending Khartoum model clinic. Ahfad Journal 1992; 9(2):23-36. |
|  | Elshibly EM, Schmalisch G. Relationship between maternal and newborn anthropometric measurements in Sudan. Pediatrics International 2009; 51(3):326-331. |
|  | Ezegwui HU, Ikeako LC, Egbuji C. Fetal macrosomia: Obstetric outcome of 311 cases in UNTH, Enugu, Nigeria. Nigerian Journal of Clinical Practice 2011; 14(3):322-326. |
|  | Ezenwaka CE, Idrisa A, Okara GC. Maternal plasma glycaemia and fetal outcome in north-eastern Nigeria. Journal of Obstetrics and Gynaecology 1998; 18(5):455-459. |
|  | Friis H, Gomo E, Nyazema N, Ndhlovu P, Krarup H, Kæstel P, et al. Maternal body composition, HIV infection and other predictors of gestation length and birth size in Zimbabwe. Br J Nutr 2004; 92(5):833-840. |
|  | Harris HE, Ellison GTH, Holliday M. Is there an independent association between parity and maternal weight gain? Ann Hum Biol 1997; 2013/01;24(6):507-519. |
|  | Hussain MA, Omololu A. Maternal nutritional status and birth weight of infants in a Nigerian village between 1966 and 1975. Nutr Rep Int 1983;27(5):1005-1011. |
|  | Kafulafula G, Moodley J. Leptin levels in the obese African parturient. Journal of Obstetrics and Gynaecology 2001;21(3):228-231. |
|  | Kafulafula GE, Moodley J, Ojwang PJ, Kagoro H. Leptin and pre-eclampsia in Black African parturients. BJOG: An International Journal of Obstetrics and Gynaecology 2002;109(11):1256-1261. |
|  | Kenyon M, Jansen AA. Weight changes during pregnancy and birthweights on Rusinga Island. Journal of Obstetrics & Gynaecology of Eastern & Central Africa 1983;2(1):20-22. |
|  | Kesa H, Oldewage-Theron W. Anthropometric indications and nutritional intake of women in the Vaal Triangle, South Africa. Public Health 2005;119(4):294-300. |
|  | Knutzen VK, Davey DA. The relationship between maternal height and weight at booking and perinatal mortality. South African Medical Journal.Suid-Afrikaanse Tydskrif Vir Geneeskunde 1977;51(19):672-675. |
|  | Kruger HS. Maternal anthropometry and pregnancy outcomes: a proposal for the monitoring of pregnancy weight gain in outpatient clinics in South Africa. Curationis 2005;28(4):40-49. |
|  | Kuti MA, Abbiyesuku FM, Akinlade KS, et al. Oral glucose tolerance testing outcomes among women at high risk for gestational diabetes mellitus. J Clin Pathol 2011;64(8):718-721. |
|  | Lawoyin TO, Oyediran AB. A prospective study on some factors which influence the delivery of low birth weight babies in a developing country. Afr J Med Med Sci 1992;21(1):33-39. |
|  | Leke RJ, Nasah BT, Mtango FD. Introduction of high risk pregnancy care in rural Cameroon: health service research approach. Journal of Obstetrics & Gynaecology of Eastern & Central Africa 1988; 7(7):7-10. |
|  | Luo B, Ma X. Risk factors for preeclampsia: a case-control study. Hypertension in Pregnancy. 2013;32(4):432-8. |
|  | Margetts BM, Rowland MGM, Foord FA, Cruddas AM, Cole TJ, Barker DJP. The relation of maternal weight to the blood pressures of Gambian children. Int J Epidemiol 1991;20(4):938-943. |
|  | Mehta S, Manji KP, Young AM, et al. Nutritional indicators of adverse pregnancy outcomes and mother-to-child transmission of HIV among HIV-infected women. The American journal of clinical nutrition 2008;87(6):1639-1649. |
|  | Mohsen MA, Wafay HA. Influence of maternal anthropometric measurements and serum biochemical nutritional indicators on fetal growth. Journal of Medical Sciences 2007;7(8):1330-1334. |
|  | Neser ML. Weight gain during pregnancy of urban Bantu women. South African medical journal = Suid-Afrikaanse tydskrif vir geneeskunde 1963;37:900-905. |
|  | Nohr EA. Maternal obesity and neonatal mortality in an African setting. The Lancet 2012; 380(9850):1292-1293. |
|  | Nwagha UI, Iyare EE, Ejezie FE, Ogbodo SO, Dim CC, Anyaehie BU. Parity related changes in obesity and some antioxidant vitamins in non-pregnant women of South-Eastern Nigeria. Nigerian journal of clinical practice 2013; 15(4): 380-384. |
|  | Nyaruhucha CN, Msimbe HP. Some factors affecting birth weights in Morogoro, Tanzania. East Afr Med J 1993;70(12):749-751. |
|  | Nyaruhucha CN, Msuya JM, Ngowi B, Gimbi DM. Maternal weight gain in second and third trimesters and their relationship with birth weights in Morogoro Municipality, Tanzania. Tanzania health research bulletin 2006;8(1):41-44. |
|  | Paul AA, Muller EM, Whitehead RG. The quantitative effects of maternal dietary energy intake on pregnancy and lactation in rural Gambian women. Trans R Soc Trop Med Hyg 1979;73(6):686-692. |
|  | Ramlal RT, Tembo M, Soko A, et al. Maternal Mid–Upper Arm Circumference Is Associated With Birth Weight Among HIV-Infected Malawians. Nutrition in Clinical Practice 2012; 27(3): 416-421. |
|  | Rasmussen KM. Endocrine disorders in pregnancy: Maternal obesity associated with neonatal death in Africa. Nature Reviews Endocrinology 2012; 8(11): 636-638. |
|  | Shaper AG. Biochemical changes, blood pressure and body build during pregnancy in African and Indian primigravidae in East Africa. Afr.Med.J 1969; 46(5):290-308. |
|  | Shirima CP, Kinabo JL. Nutritional status and birth outcomes of adolescent pregnant girls in Morogoro, Coast, and Dar es Salaam regions, Tanzania. Nutrition 2005;21(1):32-38. |
|  | Theron GB, Thompson ML. A centile chart for weight gain in pregnancy for the urban population of the Western Cape, South Africa. International Journal of Gynaecology & Obstetrics 1990 Oct;33(2):127-133. |
|  | van Middendorp D, ten Asbroek A, Bio FY, Edusei A, Meijjer L, Newton S, et al. Rural and urban differences in blood pressure and pregnancy-induced hypertension among pregnant women in Ghana. Global Health. 2013 Nov 14;9:59,8603-9-59. |
| **Missing studies** | |
|  | Abena Obama, M.T., Shasha, V.W., Fodjo, J., Bonongkaho, F., Mbede, J., Kamdom Moyo, J. Foetal macrosomia in Cameroon: prevalence, risk factors and complications. West Afr J Med. 1995;14:249–254. |
|  | Djrolo F, Megnigbeto OA, De Souza J, et al. Influence of maternal weight on pregnancy outcome in Cotonou (Benin). Journal de gynecologie, obstetrique et biologie de la reproduction 2002; 31(3): 243-247. |
|  | Fabamwo AO, Akinola OI, Obi N. Feto-Maternal Outcome in Supervised Pregnancies of the Overweight Parturient. Nigerian Journal of Clinical medicine 2010; 3(1) |
|  | Obi SN, Ejike A Obute, E. A. Pregnancy Outcome in the Obese Nigerian. Tropical Journal of obstetrics and Gynaecology 2005; 21 (1): 32-35. |

**eTable 3: Extracted outcomes** from all included studies

| **Outcome group** | **Outcomes** |
| --- | --- |
| **Prevalence** |  |
| **Labour Outcomes** | Caesarean Section (overall, elective and emergency caesarean section), indications for caesarean section, type of incision (longitudinal or pfannenstiel), complications (difficult laparotomy, difficult delivery of neonate, post-operative nausea and vomiting, estimated blood loss, post dural headache, intra-operative complications), anaesthesia technique (general, spinal, epidural, combined spinal and epidural), spontaneous vaginal delivery, instrumental delivery (vacuum/forceps), spontaneous labour, induction of labour(successful/failed), augmentation of labour; prolonged labour, perineal trauma, third or fourth degree tear, episiotomy, cephalopelvic disproportion, shoulder dystocia, epidural during labour and delivery, precipitate labour. |
| **Child outcomes** | Birth weight (low, normal, macrosomia), birth weight z-scores, birth length z-scores, head circumference z-scores, stillbirth, perinatal mortality, infant death, neonatal admission to intensive care unit and special baby care unit, birth asphyxia (5 minute and 1 minute APGAR), foetal malnutrition, birth injuries, congenital abnormality, baby stay for over 24 hours in the hospital, increased number of days stayed in the hospital. |
| **Maternal outcomes** | Wound sepsis and infection (abdominal wound and episiotomy), urinary tract infection, pre-eclampsia, pregnancy induced hypertension, eclampsia, essential hypertension, gestational diabetes mellitus, glycosuria, antepartum haemorrhage, postpartum haemorrhage, maternal mortality, retained placenta, postdate pregnancy, miscarriages, maternal admission, anaemia, malpresentation, preterm rupture of membranes, gestation duration in weeks, sickle cell disease, twin gestation, admission into intensive care, thromboembolic disease, co-morbidities (diabetes, hypertension), American medical association (AMA) health status, mallampati score, mean arterial blood pressure at 20 weeks, 28 weeks and 37 weeks, systolic and diastolic blood pressure, energy intake, haemoglobin levels, hyperemesis gravidarum, termination of pregnancy, preterm labour, malaria, retroviral disease, tuberculosis positive test. |
| **Socio-demographic and other characteristics** | Age, parity, gravidity, ethnicity, weight, body mass index, gestational age at entry, employment status of women, level of education, rural/urban dwelling, marital status, living accommodation, social class, wealth, smoking, possession of identity document by pregnant women, booked antenatal clinic, income greater than 2000 South African Rand, type of housing (formal or informal housing), access to electricity, weight gain in pregnancy (low, normal, high), knowledge of pre-pregnancy weight, at least one previous miscarriage, previous caesarean section, previous hypertension in pregnancy. |

**eTable 4: Effects of maternal obesity on labour outcomes**

| **Labour outcome** | **No of studies** | **Sample size** | | **Heterogeneity p-value** | **I^2^ statistic (%)** | **Effects Model** | **Pooled RR**  **(95% CI)** | **P value** |
| --- | --- | --- | --- | --- | --- | --- | --- | --- |
|  |  | **Obese** | **Non-obese** |  |  |  |  |  |
| Caesarean section | 10^(49,50,56,64-66,68,69,74,75)^ | 1990 | 2611 | <0.001 | 87 | Random | 2.06 [1.49, 2.85] | <0.001 |
| Caesarean section**§** | 8 ^(49,50,56,66,68,69,74,75)^ | 1565 | 1975 | 0.29 | 18 | Fixed | 1.87 [1.64, 2.12] | <0.001 |
| Instrumental /assisted delivery | 8 ^(49,50,56,64-66,68,69)^ | 1316 | 1639 | 0.07 | 46 | Random | 2.72 [1.29, 5.72] | 0.008 |
| Induction and augmentation of Labour | 3 ^(64,65,68)^ | 638 | 840 | <0.001 | 93 | Random | 2.25 [0.69, 7.31] | 0.18 |
| Induction and augmentation of Labour**§** | 2 ^(65,68)^ | 538 | 631 | 0.87 | 0 | Fixed | 1.11 [0.81, 1.52] | 0.51 |
| Prolonged labour | 3 ^(49,50,56)^ | 461 | 386 | 0.28 | 21 | Fixed | 1.79 [0.71, 4.51] | 0.22 |
| Episiotomy or perineal tear | 5 (^50,56,64,68,69)^ | 812 | 846 | <0.001 | 82 | Random | 1.45 [0.84, 2.51] | 0.18 |
| Episiotomy or perineal tear**§** | 4 ^(50,56,68,69)^ | 712 | 637 | 0.08 | 56 | Random | 1.14 [0.73, 1.77] | 0.57 |
| Cephalopelvic disproportion | 3 ^(49,50,56)^ | 461 | 386 | 0.40 | 0 | Fixed | 1.86 [0.78, 4.46] | 0.16 |

§Sensitivity analysis results

**eTable 5: Effects of maternal obesity on child outcomes**

| **Child outcome** | **No of studies** | **Sample size** | | **Heterogeneity p-value** | **I^2^ statistic (%)** | **Effects Model** | **RR**  **(95% CI)** | **P value** |
| --- | --- | --- | --- | --- | --- | --- | --- | --- |
|  |  | **Obese** | **Non-Obese** |  |  |  |  |  |
| Macrosomia | 12 ^(48-51,56,63,64,68,69,73-75)^ | 12365 | 54739 | <0.001 | 92 | Random | 2.84 [2.03, 3.99] | <0.001 |
| Macrosomia**§** | 9 ^(49,51,63,64,68,69,73-75)^ | 10804 | 46613 | 0.003 | 66 | Random | 1.83 [1.51, 2.21] | <0.001 |
| Admission to special baby care unit and intensive care unit | 4 ^(56,68,69,74)^ | 831 | 755 | 0.24 | 28 | Fixed | 1.56 [1.19, 2.06] | 0.002 |
| Low birth weight | 6 ^(49,50,56,66,68,75)^ | 1091 | 1501 | 0.63 | 0 | Fixed | 0.73 [0.53, 0.99] | 0.04 |
| Stillbirth | 5 ^(65,66,68,69,75)^ | 1104 | 1692 | 0.51 | 0 | Fixed | 1.10 [0.59, 2.03] | 0.77 |
| Perinatal mortality | 3 ^(64,69,75)^ | 600 | 1007 | 0.76 | 0 | Fixed | 2.23 [0.97, 5.13] | 0.06 |
| Birth Asphyxia | 6 ^(50,56,64,68,69,74)^ | 1085 | 1119 | 0.01 | 67 | Random | 1.79 [0.89, 3.62] | 0.10 |
| Birth Injuries | 2 ^(56,69)^ | 306 | 230 | 0.85 | 0 | Fixed | 4.67 [0.81, 26.95] | 0.09 |

§Sensitivity analysis results

**eTable 6**: Effects of maternal obesity on maternal outcomes

| **Maternal outcome** | **No of studies** | **Sample size** | | **Heterogeneity**  **p-value** | **I^2^ Statistic (%)** | **Effects Model** | **RR**  **(95% CI)** | **P value** |
| --- | --- | --- | --- | --- | --- | --- | --- | --- |
|  |  | **Obese** | **Non-obese** |  |  |  |  |  |
| Wound (caesarean section, episiotomy and genital lacerations) infection | 3 ^(50,64,69)^ | 455 | 565 | 0.38 | 0 | Fixed | 3.21 [1.28, 8.06] | 0.01 |
| Gestational Diabetes Mellitus | 6 ^(56,65,68,69,74,75)^ | 1518 | 1833 | 0.62 | 0 | Fixed | 2.42 [1.47, 3.98] | <0.001 |
| Pregnancy induced hypertension | 5 ^(56,64,65,68,75)^ | 1144 | 1568 | <0.001 | 86 | Random | 2.88 [1.46, 5.68] | 0.002 |
| Pregnancy induced hypertension**§** | 3 ^(65,68,75)^ | 888 | 1279 | 0.02 | 75 | Random | 1.59 [1.02, 2.50] | 0.04 |
| Pre-eclampsia | 9 ^(47,49,50,52,56,64,69,71,74)^ | 1354 | 1706 | 0.03 | 54 | Random | 2.19 [1.58, 3.03] | <0.001 |
| Antepartum haemorrhage | 4 ^(49,50,69,74)^ | 779 | 780 | 0.77 | 0 | Fixed | 3.67 [1.77, 7.62] | <0.001 |
| Postpartum haemorrhage | 6 ^(49,50,56,68,69,74)^ | 1136 | 1061 | 0.72 | 0 | Fixed | 1.86 [1.18, 2.92] | 0.007 |
| Maternal hospital admission | 3 ^(64,68,75)^ | 651 | 1058 | 0.57 | 0 | Fixed | 1.38 [1.21, 1.57] | <0.001 |
| Urinary tract infection | 5 ^(50,56,64,65,69)^ | 948 | 1075 | 0.11 | 46 | Fixed | 1.74 [1.05, 2.88] | 0.03 |
| Postdate pregnancy | 6 ^(50,56,65,66,69,75)^ | 1277 | 1780 | 0.56 | 0 | Fixed | 1.22 [1.01, 1.47] | 0.03 |
| Malpresentation | 3 ^(49,50,69)^ | 455 | 456 | 0.82 | 0 | Fixed | 3.01 [1.43, 6.32] | 0.004 |
| Preterm rupture of membranes | 2 ^(65,74)^ | 661 | 754 | 0.29 | 12 | Fixed | 2.88 [1.78, 4.67] | <0.001 |
| Pre-existing diabetes mellitus | 3 ^(64,50,66)^ | 384 | 681 | 0.64 | 0 | Fixed | 2.98 [1.21, 7.34] | 0.02 |
| Anaemia | 4 ^(50,64,66,69)^ | 534 | 831 | 0.003 | 79 | Random | 0.59 [0.22, 1.57] | 0.29 |
| Anaemia**§** | 3 ^(50,66,69)^ | 434 | 622 | 0.73 | 0 | Fixed | 0.90 [0.75, 1.07] | 0.23 |
| Maternal mortality | 4 ^(49,50,69,75)^ | 805 | 1104 | 0.69 | 0 | Fixed | 1.54 [0.44, 5.38] | 0.50 |
| Preterm Labour | 6 ^(50,65,66,68,69,75)^ | 1303 | 1901 | 0.30 | 18 | Fixed | 0.88 [0.74, 1.03] | 0.11 |
| Retained placenta | 2 ^(49,50)^ | 250 | 250 | 0.81 | 0 | Fixed | 0.40 [0.08, 2.04] | 0.27 |
| Chronic or essential hypertension | 4 ^(49,50,64,66)^ | 484 | 781 | 0.05 | 62 | Random | 4.43 [0.93, 21.04] | 0.06 |

§Sensitivity analysis results

**eFigure 1: Caesarean section forest plot for obese and non-obese pregnant women**


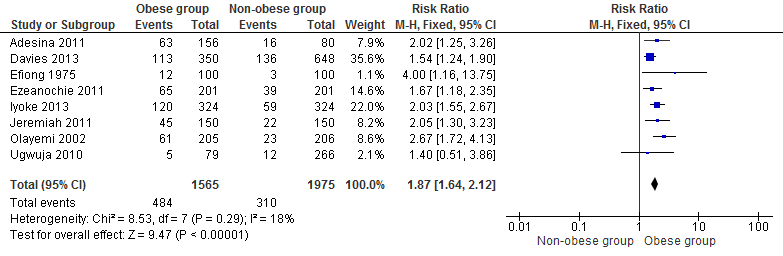


**eFigure 2: Instrumental delivery forest plot for obese and non-obese pregnant women**


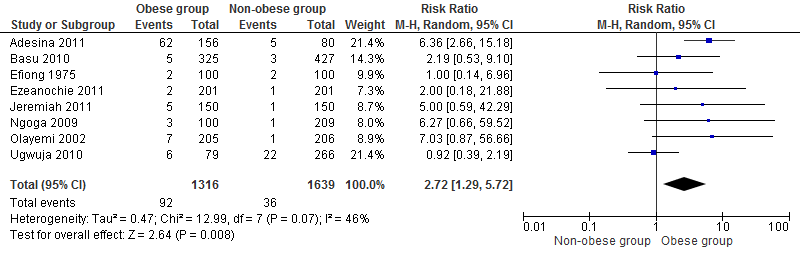


**eFigure 3: Macrosomia forest plot for obese and non-obese pregnant women**


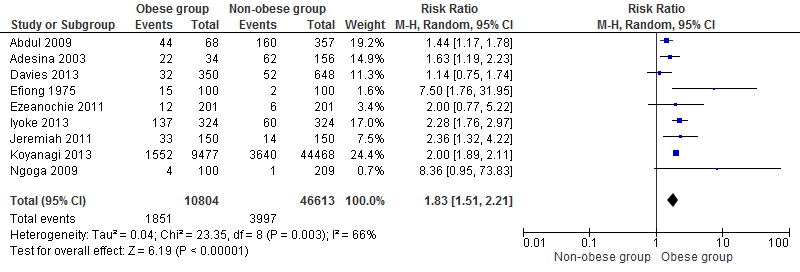


**eFigure 4: Low birth weight forest plot for obese and non-obese pregnant women**


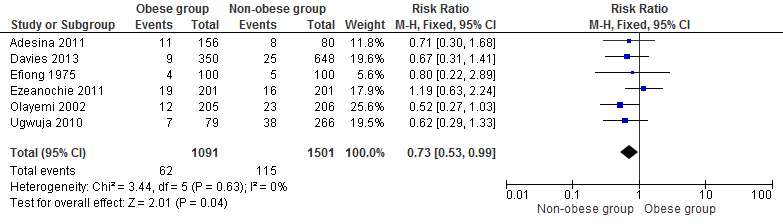


**eFigure 5: Birth Asphyxia forest plot for obese and non-obese pregnant women**


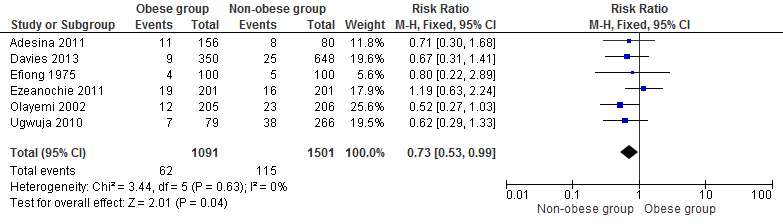


**eFigure 6: Gestational Diabetes forest plot for obese and non-obese pregnant women**


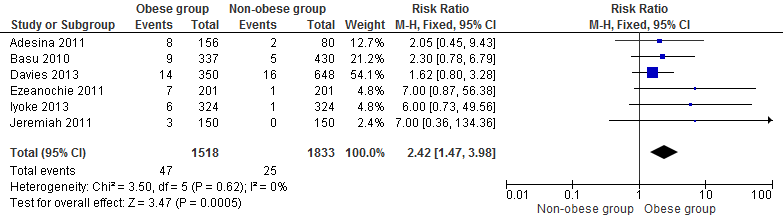


**eFigure 7: Pre-eclampsia forest plot for obese and non-obese pregnant women**


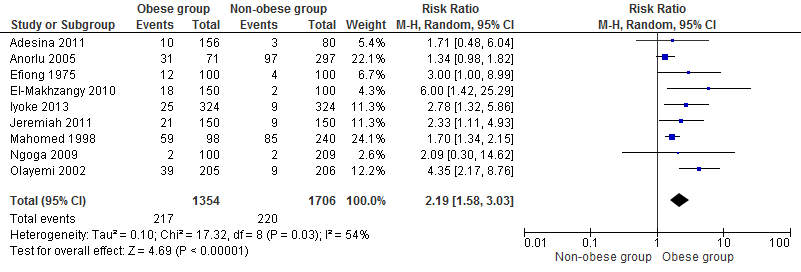


**eFigure 8: Antepartum Haemorrhage forest plot for obese and non-obese pregnant women**


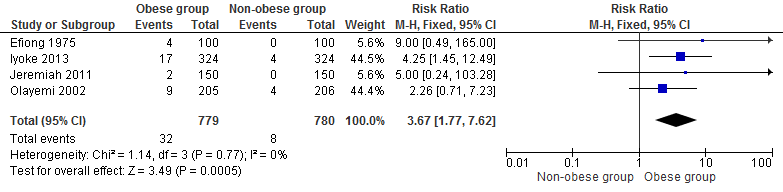


**eFigure 9: Postpartum Haemorrhage forest plot for obese and non-obese pregnant women**


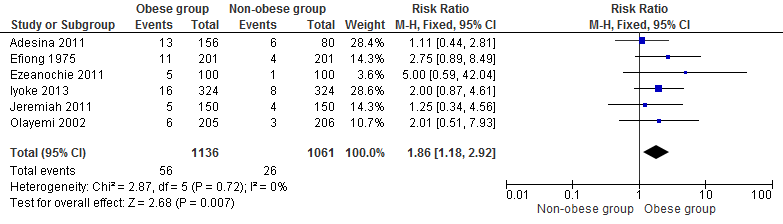

Supplement: Supplementary Data [file supp_fdv138_fdv138supp.doc]
